# Talking to migrant children and adolescents with cancer: development of a multimodal skills training in migrant-sensitive communication for paediatric oncologists

**DOI:** 10.1007/s00431-026-06787-9

**Published:** 2026-03-07

**Authors:** Anne Oommen-Halbach, Vasilija Rolfes, Dilara A. F. Vossberg, Julia Von Schreitter, Paula Merten, André Karger, Oliver Kuss, Maren Galushko, Lars Dinkelbach, Ortrun Kliche, Heiner Fangerau, Arndt Borkhardt, Prasad T. Oommen

**Affiliations:** 1 Department of the History, Philosophy and Ethics of Medicine, Medical Faculty, Heinrich Heine University Düsseldorf, Düsseldorf, Germany; 2 Department of Pediatric Oncology, Hematology and Clinical Immunology, Medical Faculty, University Hospital Düsseldorf, Heinrich Heine University Düsseldorf, Düsseldorf, Germany; 3 Clinical Institute of Psychosomatic Medicine and Psychotherapy, Medical Faculty, University Hospital Düsseldorf, Heinrich Heine University Düsseldorf, Düsseldorf, Germany; 4 Center for Integrated Oncology, Aachen Bonn Cologne Düsseldorf (CIO ABCD), Cologne, Germany; 5 German Diabetes Center, Institute for Biometrics and Epidemiology, Leibniz Institute for Diabetes Research, Heinrich Heine University Düsseldorf, Düsseldorf, Germany; 6 Centre for Health and Society, Faculty of Medicine, Heinrich Heine University Düsseldorf, Düsseldorf, Germany; 7 Cancer Counselling Centre, University Hospital Düsseldorf, Düsseldorf, Germany; 8 Department of Pediatrics II, University Hospital Essen, University of Duisburg-Essen, Essen, Germany; 9 Institute of Sex- and Gender-Sensitive Medicine, University Hospital Essen, University of Duisburg-Essen, Essen, Germany; 10 Institute for the History of Medicine and Medical Ethics, Medical Faculty, University Hospital Cologne, University of Cologne, Cologne, Germany

**Keywords:** Migrant-sensitive communication, Paediatric oncology, Communication training, Cultural humility, Shared decision-making, Medical interpreting

## Abstract

Migration remains a risk factor for unequal treatment in the healthcare system. Communication plays a key role in bridging linguistic and sociocultural differences, particularly in medically complex and ethically demanding fields such as paediatric oncology. We aim to improve the quality of intercultural conversations between paediatric patients with life-threatening diseases, their parents, and physicians. This is a monocentric, single-blind, cluster randomised interventional feasibility trial which analysed intercultural conversations to develop a skills training programme in migrant-sensitive communication for paediatric oncologists using an interventional approach. Initially, 16 conversations were observed in a non-participatory, open external observation in order to develop an observation-and interview catalogue. 10 additional observations followed by 20 interviews (10 patient families and 10 paediatric oncologists) were conducted. Based on the findings of this qualitative analysis, a literature review, and an advisory board workshop, a multimodal skills training programme in migrant-sensitive communication was developed and conducted. The observations and interview analysis identified the need for training in communication, cultural competence, and interpreter-assisted conversations. The subsequently developed skills training consisted of three modules on cultural competence, ethical issues, and interpreter-assisted conversations. The practical training included conversation simulations using three scripted patient scenarios.

*Conclusion*: Even experienced paediatric oncologists identified a need to establish ongoing training in communication in an intercultural, family-centered setting and benefited from the multimodal training.

**Table:** 

**What is Known:** *• Communication barriers in the intercultural context may lead to substantial disadvantages in healthcare.* *• Medical patient-centered communication skills can be effectively improved through training.* **What is New:** *• Migrant children with cancer are particularly affected by communication deficits resulting from medical provider uncertainty regarding handling intercultural encounters.* *• Communication training aimed at continuously improving skills and attitudes is recommended as part of specialised medical training to overcome communication barriers.*

**Supplementary information:**

The online version contains supplementary material available at 10.1007/s00431-026-06787-9.

## Introduction

Migration is a worldwide phenomenon, presenting opportunities and challenges. For individuals with a history of recent or long-standing migration, access to healthcare services is often limited, which, in turn, may lead to unequal treatment compared to people without a migration history [1, 2]. There are multifaceted reasons for the persistence of health inequalities, ranging from socioeconomic factors [3] and language barriers [4–6] to experiences of racism and discrimination in the healthcare system [7].

Migrant children and adolescents with cancer represent a particularly vulnerable group of patients. Although survival rates of childhood malignancies have increased significantly in recent decades [8–10], a paediatric cancer diagnosis remains associated with an enormous psychosocial burden on children and their families [11]. These children experience emotionally complex clinical situations that necessitate empathetic, age‑appropriate conversations that bridge linguistic and cultural barriers alike [12, 13]. In this complex setting, paediatric patients are particularly at risk of not being adequately involved and addressed in conversations with physicians.

Migrant-sensitive healthcare has recently been identified as a key area in efforts to overcome barriers to equitable healthcare for all members of society [14]. Four overarching relevant dimensions have been identified in achieving equitable healthcare: accessibility, acceptability, quality of health services, and trust in physicians [15–17]. The quality of communication between patients and physicians plays an important role in each of these dimensions. Physicians must be both willing and qualified to engage in migrant-sensitive communication with all involved parties.

Medical communication in paediatrics is characterised by a complex triadic relationship among paediatric patients, parents, and physicians, requiring both excellent communication skills among physicians and dedicated research [18]. Communication fulfills several core functions, including exchanging information, supporting decision-making, managing uncertainty, enabling patient self-management, fostering healing relationships, responding to emotions, and demonstrating solidarity and validation [19]. Successful medical communication has a positive effect on the child’s emotional and social well-being, family relationships, adherence to treatment, and therapeutic outcome [20–22]. Empirical evidence exists for the need to optimise medical conversations of physicians in this specific field and beyond [23]. The aim of this study was to identify the emotional, sociocultural, and ethical determinants of conversations and to address the complex communication requirements in paediatric oncology when patients and families with a migration history are involved.

## Methods

**Fig. 1:**
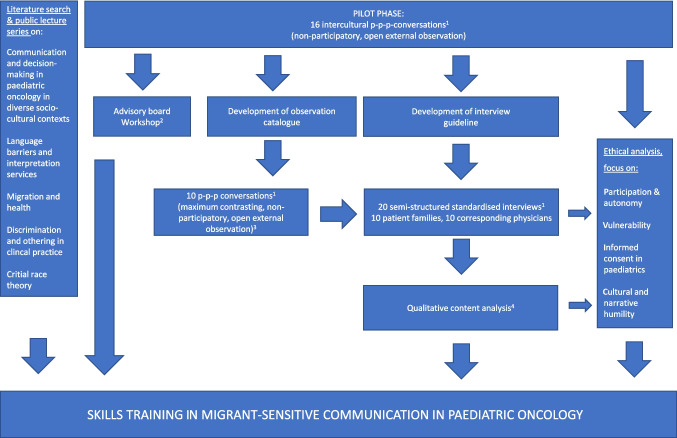
Overview of the study. ^1 ^The intercultural p-p-p-conversations in the pilot phase, the 10 maximum contrasting p-p-p-conversations later in the study, and the 20 semi-structured interviews were led by a board-certified physician in psychosomatic medicine and psychotherapy who is a qualified communication trainer and a psychologist. ^2 ^The 1-day advisory board workshop was held with a patient parent representative, a psychologist and communication trainer, a professor for pedagogy and sociology specialised on critical race theory, a medical anthropologist with experience in research projects on intercultural communication in medical settings, a professor for medical ethics, a professor for paediatric oncology, a highly experienced certified translator, a professor for biometrics, epidemiology, and health outcome research. In addition the entire research study group encompassed a member with a PhD in philosophy and medical ethics, a paediatrician with long-standing experience in medical ethics, a psychologist, a communication trainer and physician specialised in psychosomatic medicine and psychotherapy and a paediatric oncologist. ^3^Inclusion criteria for these conversations were as follows: Families with minor patients (< 18 years) and a “migration background” as defined by German Federal Statistical Office 2019 where a “migration background” exists, if a patient or at least one parent was not born a German citizen. The conversations had to be “life changing conversations” which were characterised by a lasting impact on the families’ realities, e.g. disclosure of a new oncological diagnosis, poor therapeutic response, the indication for bone marrow transplantation, or the transition to or limitation of therapy within a palliative care context.^4 ^The qualitative content analysis was conducted as described by Mayring [24]. The interviews were allocated to the following categories: conversational experience, interpreter deployment, participation of child/adolescent, handling cultural dimensions, and training needs assessment. The interviews were designed in accordance with the research design of Würth et al. [25]

The skills training described here forms the central aspect of a 3-year monocentric, single‑blind, cluster-randomised interventional feasibility trial funded by German Cancer Aid (Deutsche Krebshilfe; 70114352). This study was conducted at University Hospital Düsseldorf, Germany, and was led by an interdisciplinary team (paediatric oncology, philosophy and ethics of medicine, psycho-oncology, and biostatistics). For an overview of the process and order of the different study phases and content, see Fig. 1.

In the first part of the study, two study group members participated in 16 physician–patient–parent conversations in a non-participatory, open external observation. The study group members were a board-certified physician in psychosomatic medicine and psychotherapy who is a qualified communication trainer and a psychologist. They also participated in all following conversation observations. Only life-changing conversations, which were characterised by a lasting impact on the families’ realities, were selected. In the present study, life-changing conversations were defined as situations involving the disclosure of a new oncological diagnosis, poor therapeutic response, the indication for bone marrow transplantation, or the transition to or limitation of therapy within a palliative care context. The goal of these pilot observations was to gain insight into the field and to develop the observation catalogue and interview guideline. Of the 16 pilot conversations, there were 6 primary oncological diagnose breaking conversations, 5 conversations in preparation of a bone marrow transplant for different diagnoses, and 2 conversations in palliative care contexts. On the basis of these 16 conversations, the two above observers developed an observation catalogue and interview guideline for further non-participatory observations. These 10 additional conversations were conducted by 10 different physicians with a maximum-contrasting sample of patient families with migration history to obtain a broad picture of different needs. As in inclusion criterion we used the definition of the German Federal Statistical Office 2019 where a “migration background” exists if a patient or at least one parent was not born a German citizen [26]. Observations were documented, and subsequent semi-structured interviews were conducted with both physicians and patient families (with the children included, if possible). Each interview was recorded, transcribed, and analysed using qualitative content analysis, as described by Mayring [27]. These interviews were allocated to the following categories: conversational experience, interpreter deployment, participation of child/adolescent, handling cultural dimensions, and training needs assessment (see Table 1). The interviews were designed in accordance with the research design of Würth et al. [25].

**Table 1:** Key results from qualitative analysis

	Families	Physicians
Conversational experience	- Emotional strain in respect to life-threatening situation - High level of trust and confidence in physician - Challenges: lack of time, ambiguities, insufficient empathy, incomprehensible medical language	- Time pressure as quality limitation for conversations - Trust and honesty as basis for relationship building with patient families - Emotional stress hinders communication - Emotional assessment of families before conversations seems important
Interpreter deployment	- Very helpful to bridge language barriers - Feeling of uncertainty when language mediation is missing - Shame can hinder the use of interpreters	- Prerequisite for successful medical communication - Uncertainty as to whether interpreters are translating correctly - Misunderstandings have to be anticipated - Professional interpreters as opposed to lay interpreters are important
Participation of the child/adolescent	- Child should be integrated or the physician is to decide whether child should be present - Preference to talk to the child in a separate setting	- Age and self-assessed maturity should lead the decision whether to integrate the child or not - Child should be addressed and be the focus of the conversation - Needs, fears, and questions of the child are important - Therapy adherence can be increased when child is fully informed
Handling cultural dimension	- In general content with the way physicians dealt with specific cultural/religious issues - Cultural background of physician may be important	- Interest in training on cultural competence, cultural practices, and aspects of racism
Training needs assessment	not applicable	- High interest in further training in communication aspects - Lack of institutional support/opportunities/time in medical every-day-life

To discuss the findings of the pilot phase, such as methodological approaches and ethical considerations, a 1-day advisory board workshop was conducted in Düsseldorf, Germany, on June 13, 2023. The workshop was held with a patient parent representative, a psychologist and communication trainer, a professor for pedagogy and sociology specialised in critical race theory, a medical anthropologist with experience in research projects on intercultural communication in medical settings, a professor for medical ethics, a professor for paediatric oncology, a highly experienced certified translator and a professor for biometrics, epidemiology, and health outcome research. In addition  the entire research study group participated encompassing a member with a PhD in philosophy and medical ethics, a paediatrician with long-standing experience in medical ethics, a psychologist, a communication trainer and physician specialised in psychosomatic medicine and psychotherapy and a paediatric oncologist.

In preparation, all workshop participants had been given the same patient case in advance. This was a case study on an adolescent with a language barrier with Hodgkin Lymphoma who was being confronted with the diagnosis and somewhat disturbing side effects. This real-life case from the pilot observations covering different tasks and challenges such as participation, disclosure, and communication with a lay interpreter. Each participant was asked to give a short presentation on the case from their personal or professional perspective. The discussion focused on identifying communication deficits based on the case study and translating these into practical training content.

Ultimately, the results of the discussions in the advisory board workshop, an extensive literature search (see appendix), the public lecture series and the qualitative analysis (non-participatory, open external observation and semi-structured interviews) enhanced by an ethical analysis by the two study group members qualified in medical ethics (philosopher with PhD in philosophy and medical ethics and a board-certified paediatrician with clinical experience in paediatric oncology and ten years of experience in medical ethics teaching especially in the context of children and adolescents) led to the development of a skills training on migrant-sensitive communication. This took place at the University Hospital in Düsseldorf from February 1 to 2, 2024. Ten physicians were cluster randomised assigned to either the experimental group receiving the training or the control group (five physicians per group). Given the small sample size of physicians, the control and experimental groups were matched on key variables before randomisation to obtain balanced groups (training vs. control). All 10 participating physicians were licensed paediatricians with specialised clinical experience in paediatric oncology (median duration of clinical experience, 10.5 years; range, 1–28 years).

## Results

In the status quo interviews, all 10 paediatric oncologists identified a need for training in intercultural conversations (Table 2). One physician explicitly advocated including such training in the “mandatory curriculum of medical training” (Table 2, A).  4 out of 10 physicians highlighted the need to strengthen their communication skills. In addition to conversation techniques (B), physicians’ attitudes and mindsets (e.g. the need for more patience in intercultural conversations) were discussed. Furthermore, detailed information about the patients’ family lives was reported to be considered necessary to best tailor the conversation to each patient (C). The need for further training was particularly evident in how physicians deal with interpreters, including family members who served as substitutes for professional interpreters (D). The physicians also emphasised the complex requirements and stresses for interpreters in paediatric oncology. Thus, they recommended preparation and debriefing for the interpreters themselves (E). Furthermore, the acquisition of extended sociocultural knowledge by physicians was recommended to differentiate the religious backgrounds influencing patient families’ medical decision-making (C). Three physicians highlighted the risks of stereotyping patients, criticising the frequent use of container terms, for example, “foreign cultures” and “migration background” (F). Regarding practical exercises, the physicians indicated a need for anti-discrimination and simulation training with actors playing patients (A, G).

**Table 2:** Quotes from interviews with physicians concerning their needs in intercultural conversations (translated from German into English by PTO)

A. “This needs to be part of the compulsory curriculum. It can't just be an optional extra. It probably doesn't even need to be part of medical school, but rather part of medical practice. Because it's too early while in medical school. [...]
B. “How do you structure a conversation, how do you conduct it, what information do you share, what do you want to communicate, what should you perhaps avoid communicating? How do you respond to the parents?”
C. “So, culturally sensitive, in the sense that you educate people about these different facets. Not just, so to speak, what is religious? I think religion is something that is of high importance to many people, but it's also important to talk about the individual history of families, the topic of escape from their country and displacement, and socioeconomic status. How families who may have migrated to Germany live there. [...]”
D. "And I think that during conversations with interpreters, or rather during conversations, it should be made very clear once again how difficult it is when, for example, family members interpret. That we talk about how to do this in the most sensible way, what obstacles exist, and so on. [...]“
E. "So, specifically for interpreters, I think being an interpreter is one thing. And translating such conversations is a huge burden, and I think that afterwards, or before and after, conversations need to be held with these interpreters as well. So that they are prepared a little bit for what is coming up for them, and then afterwards, find out how they perceived it. [...]”
F. “I think discussing this concept of culture should definitely be part of regular training courses. I also think these terms that we always use are very important. I find things like “foreign cultures” sound somehow very strange. [...]”
G. "I think there is a need for anti-discrimination trainings. Because, for example, I repeatedly see patients being discriminated against because of their background and because their parents don't speak German very well, so no one even talks to them.”

### Multimodal skills training

**Fig. 2:**
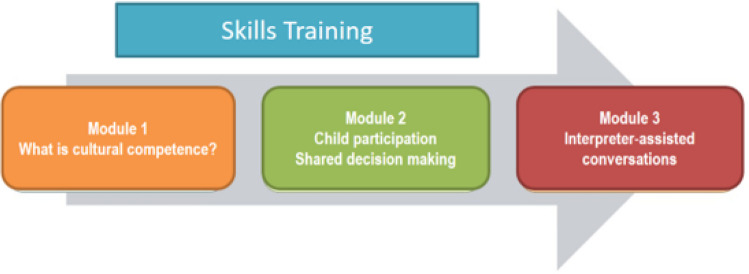
Theoretical modules of the skills training

The 2-day training course on migrant-sensitive communication comprised 12 teaching units, divided into three content modules, along with an introduction and a final discussion. Methodologically, each module consisted of a theoretical introduction and two units for practical simulations and reflection (Fig. 2, Table 3). The training was conducted by project staff with a variety of qualifications plus further experts: the introduction to the training was given by a highly experienced paediatric oncologist and paediatric palliative care specialist who is experienced in teaching on medical communication.

**Table 3:**
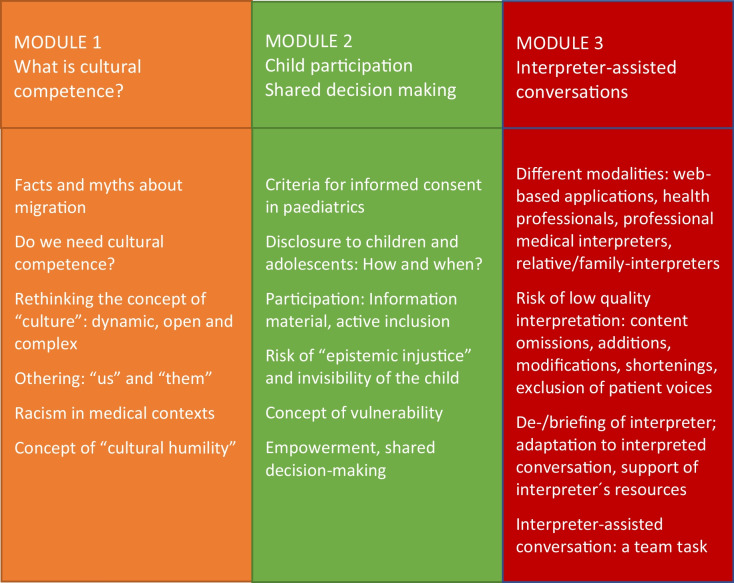
Topics of the theoretical modules: cultural competence, child participation, and interpreter-assisted conversations

Module 1 questioned the concept of cultural competence in medicine, covering facts and a critical appraisal of stereotyped thinking about migration to Germany [28]. Furthermore, the question and need for cultural competence, located on a continuum between an overemphasis on difference on the one hand and a negation of cultural differences on the other, were depicted and discussed [29, 30]. In contrast to the idea of cultural imprinting, the training focused on teaching a concept of culture as a dynamic, complex, and open process [31], leading to the challenge and necessity for physicians to take a sociocultural history more as an enquirer than as an expert. Furthermore, the topic of “othering” and the roots of racism in medicine were depicted and discussed in the context of everyday medical practice [12, 32]. Finally, the strive for cultural competence was redirected toward the central and important concept of cultural humility [33–35]. Cultural humility encompasses the idea of a physician’s attitude of modest, open-mindedness towards the individual patient and family culture. This module was led by a board-certified paediatrician with clinical experience in paediatric oncology and 10 years of experience in medical ethics teaching especially in the context of children and adolescents.

The special circumstances of children with a migration biography (e.g. language barriers and social isolation) lead to a risk that these patients will be overlooked or disadvantaged in everyday medical practice. This situation further exacerbates the issue of “epistemic injustice” [36] for this particularly vulnerable patient group. Therefore, module 2 addressed central ethical issues for paediatric patients, including criteria for informed consent, disclosure, participation, and shared decision-making [17, 37–39]. This module was therefore conducted by philosopher with a PhD in philosophy and medical ethics with 15 years of experience in medical ethics teaching and professional development.

Module 3 introduced different types of “interpreter-assisted conversations” and their challenges: on-site or remote, by multilingual health professionals, volunteer language mediators, professional medical interpreters, family members or friends, as well as web-based applications. Risks of low-quality interpreter-assisted conversations were presented including content omissions, reductions, and revisions with clinical impact and exclusion of patients’ voices. [5, 40]. Conversation-related measures focused on the physician’s skills needed to adapt to an interpreted conversation and strengthen existing resources to engage in dialogue with patient and family. Interpreting became a team task with responsibilities on all sides (e.g. physicians, interpreters, as well as institutions, and government regulations). In the practical parts of the training, physicians simulated case scenarios, which were developed and rehearsed with professional actors. These role-plays were supplemented by 360° feedback, comprising self- und peer-assessment, and were conducted by a board-certified specialist in psychosomatic medicine and psychotherapy who is a qualified communication trainer and who was additionally supported by the CoMeD-Team Düsseldorf (Communication in Medical Education Düsseldorf) with professional actors and a certified interpreter [41]. The theoretical introduction of the third module, which focused on interpreter-assisted conversations, was held by an applied linguist and translator. This trainer also led practical simulations of doctor-patient conversations, involving patient actors and a certified interpreter in the third module. The final two teaching units included a recap of the theoretical concepts which was held by a psychologist and feedback from teachers and learners regarding the methods and content of the training.

In preparation for the above scripted simulations in patient-physician interaction by professional actors, relevant and common communication models such as SPIKES (Setting, Perception, Invitation, Knowledge, Empathise, Summarise) for delivering bad news and NURSE (Naming, Understanding, Respecting, Supporting, Exploring) for handling difficult emotions were reviewed and trained followed by a constructive, multi-perspective feedback session including the participants, actors, and trainers, leading to a profound reflection on individual communication behaviour [42, 43].

## Discussion

In this interdisciplinary German Cancer Aid study, even experienced paediatric oncologists identified a wide range of training needs, from basic communication skills to specific abilities for dealing with the sociocultural differences of paediatric oncology patients, for example, the inherent risks of stereotyping and discrimination (see Table 2). General training programmes for conversations in paediatric oncology have proven necessary and successful in the past, as studies have indicated that they can improve physicians’ self-rated competence and the quality of conversations as perceived by model patients [44, 45]. While physician-.patient communication courses have reached medical schools, and medical students are exposed to mandatory simulation courses, there remains a need for experienced physicians to refresh these skills [24]. In respect to the skills training of this study, the majority of physicians provided broad positive feedback across all evaluated dimensions. The majority of the participants emphasised the clinical relevance of the role-play simulations and the feedback sessions, highlighting their value in enhancing communication skills and reflective practice. In addition, participants identified a need for simulation exercises specifically involving children and school-aged patients. They further recommended that future iterations of the training incorporate a stronger focus on discrimination, cultural sensitivity, and the sociocultural dimensions of physician–patient interactions. This was further complemented by a generally positive internal and self- and peer-assessment of the teaching staff: Critical assessment of the teaching staff encompassed the time allocation specifically for feedback rounds and a more detailed preparation of the actors in order to better simulate real-life challenges such as aggressive or difficult-to-handle patients/parents. In a recent study, a communication simulation curriculum for fellows in paediatric haematology and oncology was implemented and demonstrated to be both feasible and beneficial as a regular part of a continuous programme throughout the fellowship [46]. Some studies have shown that empathy declines even during medical training, indicating that our skills training for experienced physicians is needed and, in terms of timing, appropriately placed [47].

In recent decades, various concepts have been developed to teach “cultural competence”, especially in healthcare [48]. However, cultural assumptions about specific patient groups and their presumed customs or traditions risk promoting stereotyping, othering, and discrimination against supposedly homogeneous “foreign” patient groups [2, 15, 29, 31, 32]. Ziegler et al. identified “diversity competence” as a more general medical competence that encompasses the migration history of patients as just one aspect of a wide range of differences [30].

Furthermore, the lack of training in preparing, using, and installing professional interpreters was identified by most participating physicians as an urgent problem. In the literature, it has been shown that bridging language barriers with professional language mediators rather than lay interpreters, such as family members or mother-tongue health professionals, is often hindered by physicians’ reluctance to engage in mediated medical encounters [25, 49]. Therefore, a clear and concise overview of different techniques for preparing and meaningfully integrating professional interpreters into medical communications of physicians -which is particularly pertinent in paediatric cases-was an essential part of the training in this study.

From an ethical standpoint, the analysis revealed that children and adolescents were involved in decision-making to varying degrees, yet were largely dependent on the healthcare professional’s personal attitude. “As children’s competence is likely to reflect a dynamic construct” [50], it is influenced not only by age and cognitive skills, but also by emotional and social maturity [51]. Therefore, the extent of child and adolescent participation needs to be assessed individually [38, 50]. However, it is assumed that children’s medical decision-making capacity is much more influenced by experience than by pure knowledge [52]. In view of the life‑threatening nature of oncological diseases, the long-term medical treatment required, and the intensity and severe side-effect profile of treatment, paediatric oncology patients often gain broader experience than their healthy peers. This experience qualifies them in particular as active agents in medical decision-making processes regarding their own care and treatment [52, 53, 20]. Overall, the moral status of paediatric patients should be viewed as a delicate balance between safeguarding their well-being and respecting their developing capacity for self-determination.

Finally, the positive, non-quantifiable side effects of our study included increased sensitivity to diversity among the medical staff of the involved units. The concept of “cultural humility” was integrated into our university’s medical curriculum and subsequently presented to the public in a digital lecture series.

## Conclusion

In an increasingly diverse society, there is a growing need to train physicians in intercultural communication skills. This need is particularly relevant in life-changing conversations, where the sociocultural context of patients’ families is of substantial importance. As the individual feedback from the paediatric oncologists who participated in the study shows, the multimodal training was considered very helpful. The measurable effects will later be evaluated through a quantitative questionnaire study.

In conclusion, modular training encompassing both knowledge and skills in communication, language mediation, and basic ethical considerations is needed and should be evaluated and subsequently established as a mandatory component of ongoing specialised medical training.

## Supplementary information

Below is the link to the electronic supplementary material.

ESM 1 (PDF 103 KB)

ESM 2 (PDF 101 KB)

ESM 3 (PDF 233 KB)

## Data Availability

No datasets were generated or analysed during the current study.

## Declarations

### Registration

The study is registered under DRKS00029141 in the German Registry of Clinical Studies (DRKS).

### Data management

Data management complied with the European General Data Protection Regulation (GDPR) and the German Federal Data Protection Act. All personal data were collected, saved, and analysed pseudonymously.
