# Supplementary material for: Talking to migrant children and adolescents with cancer: development of a multimodal skills training in migrant-sensitive communication for paediatric oncologists
Source: Eur J Pediatr. 2026 Mar 7;185(3):166. doi: 10.1007/s00431-026-06787-9 (PMC12967672; doi:10.1007/s00431-026-06787-9)
Supplement: Supplementary file 1 — (PDF 103 KB) [file 431_2026_6787_MOESM1_ESM.pdf]

Interview guide for patients' families

Surname:

First name:

Age:

Place of birth:

Marital status of parents:

Nationality:

How long have you been living in Germany?

Which family member(s) came to Germany?

What language is mainly spoken in your family? (If unclear: differentiate between nuclear family, household and extended family; if several languages are spoken: which is the main one?)

What language do you read in?

Religion (practising?):

Highest level of education:

Occupation:

We are interested in hearing about your experience of the conversation with Dr. X. Please tell us about it!

Did you understand everything during the conversation? Both the language and the medical terms?

(If socially desirable answers: These were all difficult topics. I could imagine that...; if you indicate that it was difficult to understand: What was the reason you had difficulties? In your opinion, were there any difficulties beyond linguistic comprehension? What were they?)

How did you feel during the conversation?

Which part of the conversation was particularly important to you?

Was there anything in the conversation that you found very good? And anything that wasn't so good?

Was there anything in the conversation that you found unpleasant?

(If no answer: I could imagine / I had the impression that it is/was not easy to talk about ... How was that for you?)

Was there anything missing from the conversation? If so, what?

Was your family situation taken into account during the consultation? How did you notice this?

Do you now know what the next steps are for your child and your family?

Do you feel that your migration history played a role in the consultation? (In what way? How did you determine this?)

If translation aids were used

How did the translation work? (in terms of content, technology, emotion)

If no translation aids were used: would you have liked to have had a translation aid?

Do you think it is important for your child to be involved in discussions with the doctor (depending on their age)? If yes/no, why?

In your opinion, are there any other important issues that we have not addressed?

If the child participated in the conversation:

Under 12 years old

- Did you understand what it was about? What did you understand?
- How did you feel during the conversation?
- Was there anything you felt was missing from the conversation?

Over 12 years old

- Did you understand what it was about? What did you understand?
- How did you feel during the conversation?
- Did you feel well included?
- Was there anything you felt was missing from the conversation?
