# Supplementary material for: Talking to migrant children and adolescents with cancer: development of a multimodal skills training in migrant-sensitive communication for paediatric oncologists
Source: Eur J Pediatr. 2026 Mar 7;185(3):166. doi: 10.1007/s00431-026-06787-9 (PMC12967672; doi:10.1007/s00431-026-06787-9)
Supplement: Supplementary file 2 — (PDF 101 KB) [file 431_2026_6787_MOESM2_ESM.pdf]

Interview guide for physicians

Personal details

Surname:

First name:

Age:

What is your role at the clinic?

How many years of professional experience do you have in paediatric oncology?

Do you speak any languages other than German? Which ones?

Nationality:

- If not German: How long have you been living in Germany?

- If German: Nationality of parents:

Religion (practising?):

(If not clear from the observation catalogue): Did you already know the family?

We are interested in how you experienced the conversation with the amily. Please tell us about it.

How do you think the family experienced the conversation?

Did you get the impression that the family understood what you wanted to tell them, both medically and linguistically? PAUSE. How did you notice this?

I imagine that sometimes you first have to find out how best to communicate with someone. How was that for you with this family?

Did you experience any social or cultural peculiarities in the conversation that went beyond linguistic communication? What were they? (If nothing comes to mind, but there was something: I noticed that...)

Was there anything that struck you as particularly positive during your conversation with the family?

Were there any moments when you had the impression that it was particularly difficult or unpleasant for the family? How did you notice that?

*If the child participated in the conversation:*

• How did you feel about the child participating in the conversation?

• How was it decided in advance whether and how the child should be involved?

• Do you think they felt well integrated?

• Were there any indications that the child found other things important than the parents and wanted to discuss them?

In your opinion, what should determine whether a child participates in the conversation or not?

*If interpreters or language and integration mediators were used:*

• How did it come about that an interpreter or language and integration mediator was used?

• How did you experience the situation?

If linguistic communication with a family is not possible, how do you proceed?

Do you feel well prepared to deal with patients who have difficulties with the English language or come from a different cultural background?

What is the biggest challenge for you in this context?

Is there anything beyond what has been discussed so far that you regularly notice when encountering patients who do not speak German well or who are culturally different from you?

We often talk about cultural differences in this context, but it often remains unclear what exactly is meant by this. What do you understand by cultural differences?

Do you see any room for improvement in the organisation of the outpatient clinic or ward that could facilitate communication with patients who have language difficulties?

Do you see a need for further training on the topics of 'culturally sensitive communication' or 'communicating with interpreters'? If so, what kind?

What would you expect from training on the topic of 'culturally sensitive communication' – apart from interpreter support?

Do you find out about the cultural backgrounds of the people you are talking to in advance? How does the way you conduct conversations with families with and without a migration background differ for you?
