# Supplementary material for: Talking to migrant children and adolescents with cancer: development of a multimodal skills training in migrant-sensitive communication for paediatric oncologists
Source: Eur J Pediatr. 2026 Mar 7;185(3):166. doi: 10.1007/s00431-026-06787-9 (PMC12967672; doi:10.1007/s00431-026-06787-9)
Supplement: Supplementary file 3 — (PDF 233 KB) [file 431_2026_6787_MOESM3_ESM.pdf]

Appendix

(1) communication in (pediatric) oncology and decision-making processes in pediatric oncology, with focus on diverse socio-cultural contexts

Back AL, Arnold RM, Baile WF, Tulsky JA, Fryer-Edwards K (2005*)* Approaching difficult communication tasks in oncology. *CA:* A Cancer Journal for Clinicians 55(3):164-177. DOI: 10.3322/canjclin.55.3.164

Becktell K, Rumler S, Tower RL. Successful Development and Implementation of Communication Skills Simulation Training Within Pediatric Hematology-oncology Fellowship Education. J Pediatr Hematol Oncol. 2025 Jan 1;47(1):7-11. doi: 10.1097/MPH.0000000000002959. Epub 2024 Oct 17. PMID: 39526965

Bell J, Condren M. Communication Strategies for Empowering and Protecting Children. J Pediatr Pharmacol Ther. 2016 Mar-Apr;21(2):176-84. doi: 10.5863/1551-6776-21.2.176. PMID: 27199626; PMCID: PMC4869776

Bluebond-Langner M, Nordquest Schwallie M, "It's Back": Children with Cancer Talking about Their Illness When Cure is Not Likely. In: Comacchio C, Golden J, Weisz G (Eds), Healing the World's Children. Interdisciplinary Perspectives on Child Health in the Twentieth Century, Montreal & Kingston, London, Ithaca: McGill-Queen's University Press 2008, 161-175

Chittem M, P. Butow P, Responding to family requests for nondisclosure: the impact of oncologists' cultural background. J Cancer Res Ther. 11,1 (2015):174-80. doi: 10.4103/0973-1482.140836

Dietrich K, Leiss U, Griessmeier B, Wiener A, Minetzke-Gruner AC, Linhart D, Braungart R, Graf N, Wevers-Donauer G, Parental Distress Caused by Child´s Cancer Diagnosis. *Klin Padiatr* 2016;228(03):149-156

Dimatteo MR. The role of effective communication with children and their families in fostering adherence to pediatric regimens. Patient Educ Couns. 2004 Dec;55(3):339-44. doi: 10.1016/j.pec.2003.04.003. PMID: 15582339

Feudtner C, Walter JK, Faerber JA, Hill DL, Carroll KW, Mollen CJ, Miller VA, Morrison WE, Munson D, Kang TI, Hinds PS (2015) Good-parent beliefs of parents of seriously ill children. JAMA Pediatr 169:39–47

Harris VC, Links AR, Walsh J, Schoo DP, Lee AH, Tunkel DE, Boss EF (2018) A systematic review of race/ethnicity and parental treatment decision-making. Clin Pediatr (Phila) 57:1453-1464. doi: 10.1177/0009922818788307

Hinds PS, Oakes LL, Hicks J, Powell B, Srivastava DK, Spunt SL, Harper J, Baker JN, West NK, Furman WL (2009) Trying to be a good parent as defined by interviews with parents who made phase I, terminal care, and resuscitation decisions for their children. J Clin Oncol 27:5979–5985

Mack JW, Cronin AM, Kang TI (2016) Decisional regret among parents of children with cancer. J Clin Oncol 34:4023–4029

Sisk BA et al. (2020) The evolution of regret: decision-making for parents of children with cancer, in: Support Care Cancer 28; 3:1215-1222

Sisk BA, Bluebond-Langner M, Wiener L, Mack J, Wolfe J (2016) Prognostic Disclosures to Children: A Historical Perspective. Pediatrics 138(3):e20161278

Sisk BA, Friedrich AB, Blazin LJ, Baker JN, Mack JW, DuBois J (2020) Communication in Pediatric Oncology: A Qualitative Study. Pediatrics 146(3):e20201193

Sisk BA, Friedrich AB, Mozersky J, Walsh H, DuBois J (2020) Core Functions of Communication in Pediatric Medicine: an Exploratory Analysis of Parent and Patient Narratives. J Cancer Educ 35(2):256-263. doi: 10.1007/s13187-018-1458-x

Sisk BA, Harvey K, Friedrich AB, Antes AL, Yaeger LH, Mack JW, Dubois JM (2021) Multilevel barriers and facilitators of communication in pediatric oncology: A systemativ review 69(1):e29405.doi 10.1002/pbc.29405

Sisk BA, Kang TI, Goldstein R, et al.: Decisional burden among parents of children with cancer. Cancer 2019;125: 1365–1372

Sisk BA, Kang TI, Mack JW (2020) Racial and Ethnic Differences in Parental Decision-Making Roles in Pediatric Oncology. J Palliat Med. 23,2 (2020):192-197. doi: 10.1089/jpm.2019.0178

Sisk BA, Schulz GL, Blazin LJ, Baker JN, Mack JW, Dubois JM (2021) Parental views on communication between children and clinicians in pediatric oncology: a qualitative study 29(9): 4957-4968.doi: 10.1007/s00520-021-06047-6

Sisk BA, Schulz GL, Kaye EC, Baker JN, Mack JW, Dubois JM (2021) Clinicians' Perspectives on the Functions of Communication in Pediatric Oncology 24 (10):1545-1549. doi: 10.1089/jpm.2021.0090

Stein A, Dalton L, Rapa E, Bluebond-Langner M, Hanington L, Stein KF, Ziebland S, Rochat T, Harrop E, Kelly B, Bland R (2019) Communication Expert Group. Communication with children and adolescents about the diagnosis of their own life-threatening condition. Lancet 16,393:1150-1163

Wiener L., D.G. McConnell, L. Latella, E. Ludi, Cultural and religious considerations in pediatric palliative care. Palliat Support Care 11,1 (2013):47-67. doi: 10.1017/S1478951511001027

Zolnierek KB, Dimatteo MR. Physician communication and patient adherence to treatment: a meta-analysis. Med Care. 2009 Aug;47(8):826-34. doi: 10.1097/MLR.0b013e31819a5acc. PMID: 19584762; PMCID: PMC2728700

(2) communication interventions aiming at enhancing communication in pediatric oncology and/or in the intercultural context

Feraco AM, Brand SR, Mack JW, Kesselheim JC, Block SD, Wolfe J (2016) Communication skills training in paediatric oncology: moving beyond role modeling. Paediatric blood & cancer 63(6): 966-972

Govere L, Govere EM (2016) How Effective is Cultural Competence Training of Healthcare Providers on Improving Patient Satisfaction of Minority Groups? A Systematic Review of Literature. Worldviews Evid Based Nurs 13(6):402-410. https://doi.org/10.1111/wvn.12176

Karger A, Petermann-Meyer A, Vitinius F, Geiser F, Marx A, Ernstmann N, Ernsten L, Röhlinger M, Buller S, Panse J (2023) KommRhein Interpro Manual für fertigkeitenorientierte, interprofessionelle Kommunikationstrainings in der Onkologie mit Teams aus Ärztinnen, Ärzten und Gesundheits- und Krankenpflegenden (GKP) https://doi.org/10.24336/hhubooks.42

Kaye EC, Cannone D, Snaman JM, Baker JN, Spraker-Perlman H, The state for the science for communication training in paediatric oncology: A systemativ review. Paediatric Blood & Cancer 67 (2020):e28607. doi: 10.1002/pbc.28607

Luttenberger K, Graessel E, Simon C, Donath C (2014) From board to bedside - training the communication competences of medical students with role plays. BMC Med Educ 14:135. doi: 10.1186/1472-6920-14-135. PMID: 24996804; PMCID: PMC4096752

Ranmal R, Prictor M, Scott JT. Interventions for improving communication with children and adolescents about their cancer. *Cochrane Database Syst Rev*. 2008(4):CD002969

Schouten B, Meeuwesen L (2006) Cultural differences in medical communication: A review of the literature. Patient Educ Couns 64 (1-3):21-34

Sisk BA, Schulz GL, Mack JM, Yaeger L, DuBois J (2019) Communication interventions in adult and pediatric oncology: A scoping review and analysis of behavioral targets, Plos One 22;14(8):e0221536. doi: 10.1371/journal.pone.0221536

(3) migration and health in respect to language barriers and interpretation services

Abbe M, Simon C, Angiolillo A, Ruccione K, Kodish ED (2006) A survey of language barriers from the perspective of pediatric oncologists, interpreters, and parents, in: Pediatr Blood Cancer 2006;47:819-824

Bermejo I, Hölzel L, Kriston L, Härter M (2012) Subjektiv erlebte Barrieren von Personen mit Migrationshintergrund bei der Inanspruchnahme von Gesundheitsmaßnahmen. Bundesgesundheitsblatt 55, 944-953

Diamond LC, Schenker Y, Bradley EH, Fernandez A (2009) Getting By: Underuse of Interpreters by Resident Physicians. Journal of General Internal Medicine 24 (2): 256-262

Führer A, Brzoska P (2022) Die Relevanz des Dolmetschens im Gesundheitssystem, in: Gesundheitswesen 84:474-478

Führer A, Taché S, Riemenschneider H, Bozorgmehr K, Diaz-Monsalve S, Knipper M, Mews C, Schwienhorst-Stich EM, Siebert U, Strelow KU, Ziegler S, Das Lehrnetzwerk Migration und Gesundheit: Aus- und Weiterbildung konsolidieren und weiterentwickeln. Bundesgesundheitsbl 66 (2023):1130-1134

Führer A, Tiller D, Brzoska P, Korn M, Gröger C, Wienke A (2019) Health-Related Disparities among Migrant Children at School Entry in Germany. How does the Definiton of Migration Status matter? Int J Environ Res Public Health 17:212; doi: 10.3390/ijerph17010212

Jungner JG, Tiselius E, Blomgren K, Lützén K, Pergert P (2019) The interpreter's voice: Carrying the bilingual conversation in interpreter-mediated consultations in pediatric oncology care. Patient Education and Counseling, 102(4), 656-662

Kliche O, Güzelsoy L (2025) Ethik ohne Worte? Wie Sprachbarrieren und Vorannahmen die Selbstbestimmung von Patient:innen untergraben. Ethik Med 37, 75–77. https://doi.org/10.1007/s00481-025-00849-6

Langer T, Unseld A, Tošic R, Meyer B (2024) Die stille Hürde überwinden – Effektiv und kompeten mit Dolemtschenden zusammenarbeiten. Monatsschrift Kinderheilkunde 172:154-160

Rakic M, Sandri A, Gysin O, Rost M (2025) Migrant families' experiences in pediatric oncology: A scoping review. Eur J Oncol Nurs. 2025 Oct;78:102948. doi: 10.1016/j.ejon.2025.102948. Epub 2025 Aug 5. PMID: 40865365

Razum O, Karrasch L, Spalek J (2016) Migration. Eine vernachlässigte Dimension gesundheitlicher Ungleichheit. Bundesgesundheisbl 59(2):259-265

Savas S, Knipper M, Duclos D, Sharma E, Ugarte-Gurrutxaga MI, Blanchet K (2024) Migrant-sensitive healthcare in Europe: advancing health equity through accessibility, acceptability, quality, and trust, The Lancet Regional Health-Europe 41: 100805, https://doi.org/10.1016/j.lanepe.2023.100805

Savas S, Knipper M, Duclos D, Sharma E, Ugarte-Gurrutxaga MI, Blanchet K (2024) Migrant-sensitive healthcare in Europe: advancing health equity through accessibility, acceptability, quality, and trust, The Lancet Regional Health-Europe 41: 100805, https://doi.org/10.1016/j.lanepe.2023.100805

Würth K, Langewitz W, Reiter-Theil S, Schuster S (2018) Their view: difficulties and challenges of patients and physicians in cross-cultural encounters and a medical ethics perspective, BMC Medical Ethics 19:70, https://doi.org/10.1186/s12910-018-0311-4

Würth K, Reiter-Theil S, Langewitz W, Schuster S (2018), „Getting by" in a Swiss Tertiary Hospital: the Inconspicuous Complexity of Decision-making Around Patients' Limited Language Proficiency, Journal of General Internal Medicine, https://doi.org/10.1007/s11606-018-4618-0

(4) racism, discrimination, and othering in clinical practice

Akbulut N, Razum O (2023) Othering am Beispiel von Migration: Wie aus sozialen Kategorien die Anderen entstehen. Bundesgesundheitsblatt 66;1109-1116

Driesch, Georg, Wenn das Ich das Fremde trifft, in: Gillessen, A, Golsabahi-Broclawski S., Biakowski A., Broclawski A (Hrsg): Interkulturelle Kommunikation in der Medizin, Heidelberg: Springer 2020, 27-38

Führer A, Tiller D, Brzoska P, Korn M, Gröger C. and Wienke A (2020) Health-related disparities among migrant children at school entry in Germany. How does the definition of migrationstatus matter? Int JEnviron Res Public Health 17:212. https://doi.org/10.3390/ijerph17010212

Kajikhina K, Koschollek C, Bozorgmehr K, Sarma N, Hövener C (2023) Rassismus und Diskriminierung im Kontext gesundheitlicher Ungleichheit – ein narratives Review. Bundesgesundheitsbl 66:1099-1108

Kajikhina K, Koschollek C, Sarma N et al (2023) Recommendations for collecting and analysing migration-related determinants in public health research. J HealthMonit 8:52–72. https://doi.org/10.25646/11144

Karger, André, Heide Lindtner-Rudolph, Robert Mroczynski, Alexander Ziem, Ljiljana Joksimovic, „Wie fremd ist mir der Patient?“ Erfahrungen, Einstellungen und Erwartungen von Ärztinnen und Ärzten bei der Versorgung von Patientinnen und Patienten mit Migrationshintergrund, in: Zeitschrift für Psychosomatische Medizin und Psychotherapie 63 (2017) No. 3, 280-296

Knipper M (2013) Migrationshintergrund? Plädoyer für eine zeitgemäße Beachtung der sozialen und kulturellen Hintergründe von Kindergesundheit in Deutschland. In: Bundesverband der Kinder und Jugendärzte e. V. (Hrsg) Schwerpunktthema Migrantinnen und Migranten in der Pädiatrie, BVKJ,K.ln, S65–69

Will A-K (2016) 10 Jahre Migrationshintergrund in der Repräsentativstatistik: ein Konzept auf dem Prüfstand. Leviathan 44(1):9–35

Will, Anne-Kathrin (2019) The German statistical category “migration background”: Historical roots, revisions and shortcomings. Ethnicities 19 (3):535-557

(5) cultural humility and narrative humility, as well as training programs on intercultural aspects for physicians.

Carillo, J. Emilio, Alexander R. Green, Jospeh R. Betancourt, Cross-Cultural Primary Care: A Patient-Based Approach, in: Annals of Internal Medicine 130 (1999) 829-834

Cross T, Bazron B, Dennis K, et al (1989) Towards a culturally competent system of care. Georgetown University Child Development Center: CASSP Technical Assistance Center

DasGupta S (2008) Narrative humility. Lancet 371:980–981. https://doi.org/10.1016/S0140-6736(08)60440-7

Gray WN, LJ Szulczewski, SM Regan, JA Williams, AL. Pai, Cultural influences in pediatric cancer from diagnosis to cure/end of life. J Pediatr Oncol Nurs. 31,5 (2014):252-71. doi: 10.1177/1043454214529022

Greene-Moton E, M. Minkler, Cultural Competence or Cultural Humility? Moving Beyond the Debate. Health Promot Pract 21,1 (2020):142-145. doi: 10.1177/1524839919884912

Grützmann, T., Interkulturelle Kompetenz in der klinisch-ethischen Praxis. Kultursensible Ansätze zum Umgang mit interkulturellen Situationen in der klinischen Ethikberatung, Berlin und Münster: LIT-Verlag 2016

Hook JN (2014) Engaging clients with cultural humility. J Palliat Care 33:277–280

Kirmayer LJ (2012) Rethinking cultural competence. Transcult Psychiatry 49:149–164. https://doi.org/10.1177/1363461512444673

Kleinman A, Benson P (2006) Anthropology in the Clinic: The Problem of Cultural Competency and How to Fix It, PLoS Med 3(10): e294, https://doi.org/10.1371/journal.pmed.0030294

Knipper M, Orcutt M, Duclos D, Hanewald B, Blanchet K (2022) Cultural Competency and cultural adjustment of services. In: Orcutt N, Shortall C, Walpole S, et al. (eds) Handbook of refugee health: For healthcare professionals and humanitarians providing care to forced migrants, CRC Press:59-65

Mecheril, P., „Kompetenzlosigkeitskompetenz“. Pädagogisches Handeln unter Einwanderungsbedingungen, in: Interkulturelle Kompetenz und pädagogische Professionalität, hrsg. von G. Auernheimer, Opladen: Leske + Budrich 2008, 15-34

Murray-García J.L., V. Ngo, T.A. Yonn-Brown, D.H. Hosley, H. Ton, California's Central Valley: Teaching Social Determinants of Health and Cultural Humility Through an Interprofessional, Overnight Road Trip. J Health Care Poor Underserved. 33,2 (2022):819-841

Peters, T., T. Grützmann, W. Bruchhausen, M. Coors, F. Jacobs, L. Kaelin, M. Knipper, F. Kressing und G. Neitzke, Grundsätze zum Umgang mit Interkulturalität in Einrichtungen des Gesundheitswesens, in: Ethik in der Medizin 26,1 (2014):65-75

Roncoroni J., C.M. Tucker, W. Wall, K. Nghiem, R.S. Wheatley, W. Wu, Patient Perceived Cultural Sensitivity of Clinic Environment and Its Association With Patient Satisfaction With Care and Treatment Adherence. American Journal of Lifestyle Medicine 8,6 (2014):421-429. doi:10.1177/1559827614521760

Shepherd SM (2019) Cultural awareness workshops: limitations and practical consequences. BMC Med Educ 19:14. https://doi.org/10.1186/s12909-018-1450-5

Tervalon M., J. Murray-Garcia, Cultural humility versus cultural competence: a critical distinction in defining physician training outcomes in multicultural education, in: J Health Care Poor Underserved 9 (1998):117-125

Watt, Kelly, Penny Abbott and Jenny Reath, Developing cultural competence in general practitioners: an integrative review of the literature, in: BMC Family Practice 17 (2016):158, DOI 10.1186/s12875-016-0560-6

Ziegler S, Michaëlis C, Sørensen J (2022) Diversity Competence in Healthcare: Experts' Views on the Most Important Skills in Caring for Migrant and Minority Patients. Societies 12,43, https://doi.org/10.3390/soc12020043

(6) autonomy and informed consent among pediatric patients

American Academy of Pediatrics. Committee on Bioethics (2016) Policy statement: informed consent in decision-making in pediatric practice. Pediatrics 138:e20161484. https://doi.org/10.1542/peds.2016-1485

Archard D (2015) Children, adults, autonomy and well-being. In: Bagattini A, Macleod C (eds) The nature of children's well-being: theory and practice. Springer, Dordrecht

Badarau DO, De Clercq E, Wangmo T, Dragomir M, Miron I, Kühne T, Elger BS (2016) Cancer care in Romania: challenges and pitfalls of children's and adolescents' multifaceted involvement. J Med Ethics 42:757–761. https://doi.org/10.1136/medethics-2016-103418

Hein IM, Troost PW, Broersma A, de Vries MC, Daams JG, Lindauer RJL (2015) Why is it hard to make progress in assessing children's decision-making competence? BMC Medical Ethics 16 (1), http://www.biomedcentral.com/1472-6939/16/1

Hein, IM, P.W. Troost, R. Lindeboom, M.A. Benninga, C.M. Zwaan, J.B. van Goudoever, R.J.L. Lindauer, Accuracy of the MacArthur competence assessment tool for clinical research (MacCAT-CR) for measuring children's competence to consent to clinical research, in: JAMA pediatrics 168 (12):1147-1153

Ho A (2006) Family and informed consent in multicultural setting. Am J Bioethics 6:26–28. https://doi.org/10.1080/15265160600715753

McCabe MA (1996) Involving children and adolescents in medical decision making: developmental and clinical considerations. J Pediatr Psychol 21:505–516. https://doi.org/10.1093/jpepsy/21.4.505

Mengel, R., F. Wedemeyer, C. Wiesemann, Partizipation von Kindern und Jugendlichen im Krankenhaus. frühe kindheit 22 (2019) 20-28

Michaud, PA, Blum R, Benaroyo L, Zermatten J, Baltag V (2015) Assessing an Adolescent's Capacity for Autonomous Decision-Making in Clinical Care, Journal of Adolescent Health 57: 361-366

Oommen-Halbach A, Fangerau H (2019) Selbstbestimmung von Kindern in der Medizin. In: Drerup J, Schweiger G (eds) Handbuch Philosophie der Kindheit. JB Metzler, Stuttgart. https://doi.org/10.1007/978-3-476-04745-8_35

Pyke-Grimm KA, Kelly KP, Schulz GL (2025) Child and adolescent cancer communication preferences for treatment decision making: a meta-synthesis. Pediatr Blood Cancer 72:e31944. https://doi.org/10.1002/pbc.31944

Wiesemann C (2016) Moral equality, bioethics, and the child. Springer, Cham

(7) lack of involvement of children and young people (regardless of migrant status)

Sisk BA, Mack JW, Ashworth R, DuBois J (2018) Communication in pediatric oncology: state of the field and research agenda. Pediatr Blood Cancer 65: e26727. https://doi.org/10.1002/pbc.26727

Sisk BA, Newman AR, Chen D, Mack JW, Reeve BB (2023) Designing and validating novel communication measures for pediatric, adolescent, and young adult oncology care and research: the PedCOM measures. Pediatr Blood Cancer 70: e30685. https://doi.org/10.1002/pbc.30685

Snaman JM, Helton G, Holder RL, Wittenberg E, Revette A, Tulsky JA, et al (2021) MyPref: pilot study of a novel communication and decision-making tool for adolescents and young adults with advanced cancer. Support Care Cancer 29: 2983–2992. https://doi.org/10.1007/s00520-020-05806-1
